# A Partial Frontal Fracture of the Humeral Trochlea: A Case Report

**DOI:** 10.7759/cureus.56640

**Published:** 2024-03-21

**Authors:** Aboubacar Lawan Abdou, Taha El aissaoui, Adnane Lachkar, Najib Abdeljaouad, Hicham Yacoubi

**Affiliations:** 1 Department of Traumatology and Orthopedics, Mohammed VI University Hospital, Oujda, MAR

**Keywords:** joint function, rehabilitation, herbert screw, surgical treatment, elbow, trochlea, humeral fracture

## Abstract

Fractures of the lower end of the humerus are uncommon but serious, potentially compromising elbow function. This article reports the case of a young patient with a fracture of the inner cheek of the humeral trochlea, resulting from a public road accident. The diagnosis was established by radiography and CT scan, confirming a displaced fracture associated with an avulsion fracture of the coronoid process. Surgical treatment was carried out with fixation of the osteochondral fragment and evacuation of the intra-articular fragments. Two months after the operation, the patient regained good joint function with a resumption of professional activity.

## Introduction

Fractures of the distal end of the humerus are relatively rare, accounting for 2% of all body fractures [1]. They are serious and can compromise the functional prognosis of the elbow. Isolated trochlear fractures were first described by Laugier in 1853 and then by Stimson. They are thus referred to by some authors as Laugier's fractures [2]. Their management requires a systematic approach, anatomical reduction of the articular surface by stable fixation, allowing for immediate postoperative active mobilization [3].

We report a particular case of a frontal fracture of the inner edge of the humeral trochlea in a young patient.

## Case presentation

The patient is a 23-year-old male with no known medical history, who was involved in a road traffic accident involving a collision between two motorcycles, with a reported fall onto the palm of the right hand, elbow in hyperextension, resulting in pain and total functional impairment of the right elbow.

Clinical examination on admission revealed a patient with a typical traumatic upper limb posture, with the left upper limb supporting the injured right limb. The right elbow was globally swollen with loss of bony landmarks, but no open wound was observed. All movements were impossible due to pain. Vascular and neurological examinations were normal.

**Figure 1:**
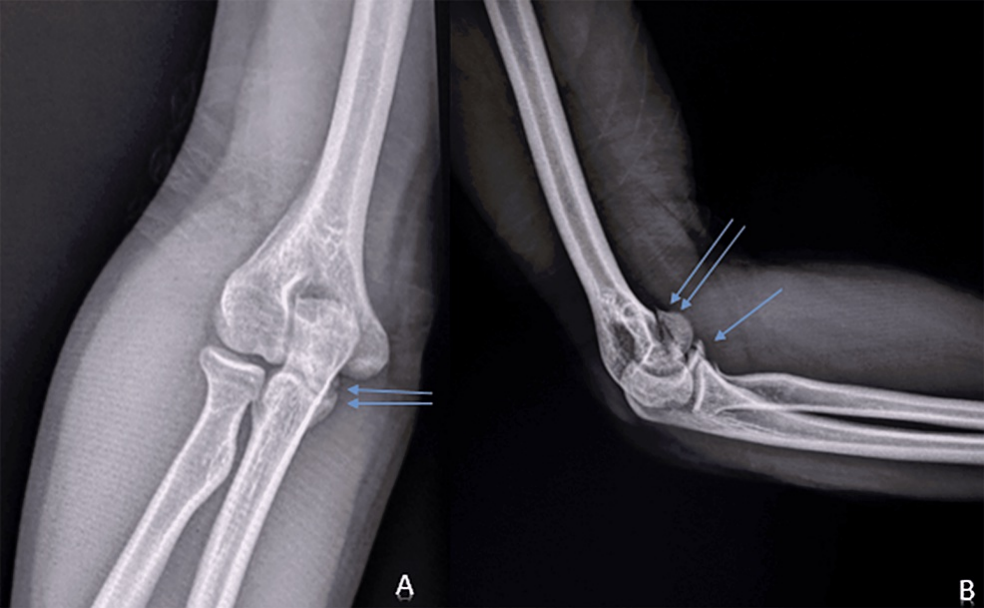
Standard radiograph in anteroposterior (A) and lateral (B) views showing a fracture of the humeral trochlea (two blue arrows) and avulsion of the coronoid process (one blue arrow)

**Figure 2:**
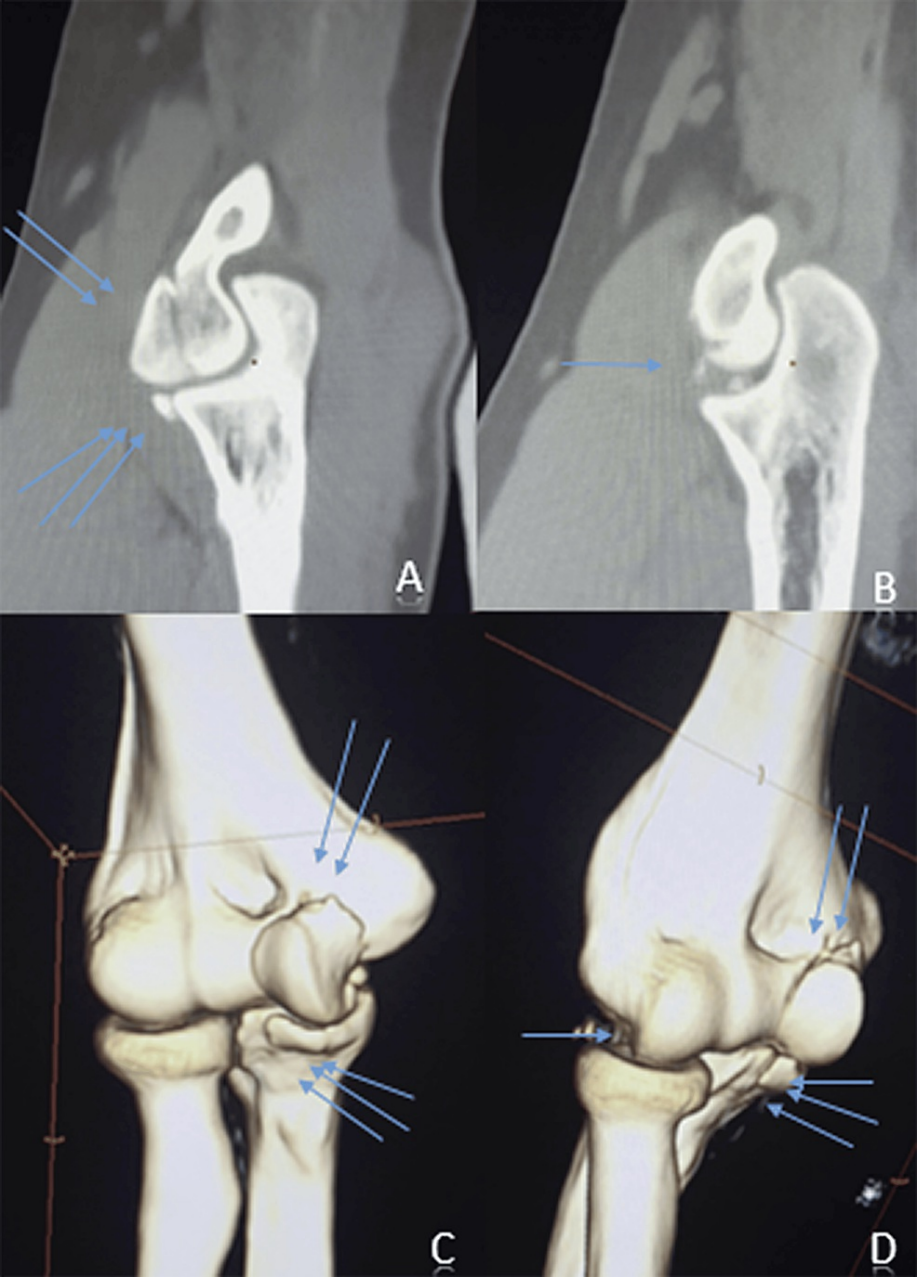
CT scans of the elbow. A and B (sagittal sections), C and D (3D reconstructions) showing intra-articular fragments (one blue arrow), the trochlear fracture (two blue arrows), and avulsion of the coronoid process (three blue arrows)

The patient underwent radiological assessment, including standard radiographs of the right elbow in anteroposterior and lateral views (Figure 1), supplemented by computed tomography (Figure 2). The diagnosis of a displaced fracture of the inner edge of the humeral trochlea associated with a type I coronoid process avulsion fracture, according to Regan and Morrey classification, was established. Standard radiographs showed irregularity of the articular surface and a characteristic half-moon appearance. The capitulum was intact.

**Figure 3:**
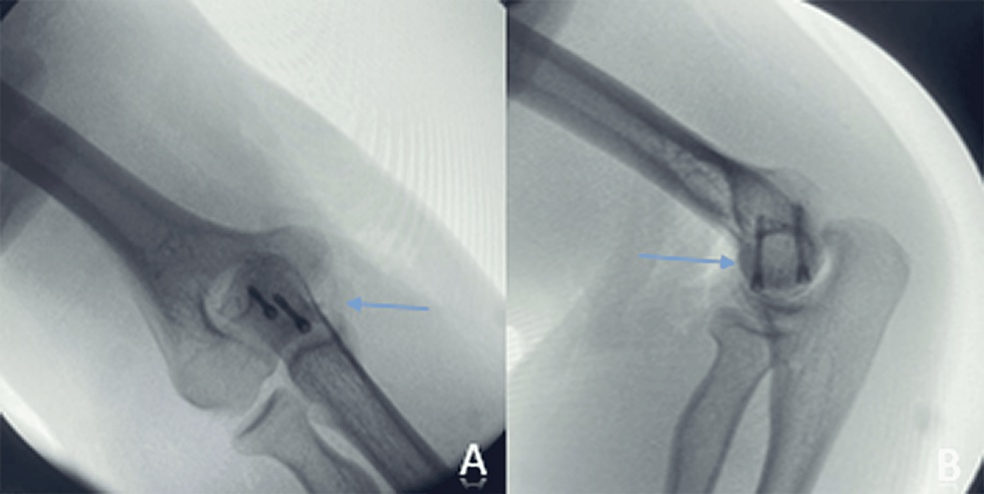
Intraoperative fluoroscopic images in anteroposterior (A) and lateral (B) views showing the placement of the two Herbert screws (blue arrow)

Our therapeutic decision was to proceed with surgical treatment. Through an anteromedial approach, we performed fixation of the single osteochondral fragment using two Herbert screws, buried intra-articularly (Figure 3), ensuring the emptiness of the olecranon fossa. The reduction was satisfactory under direct visualization and fluoroscopic control. Intra-articular fragments, visualized on the CT scan, were evacuated by pressurized saline lavage and fluoroscopic control. We decided not to intervene in the coronoid process due to the small size of the fragment and the preserved stability of the elbow. Closure was performed without a Redon drain according to our routine practice.

**Figure 4:**
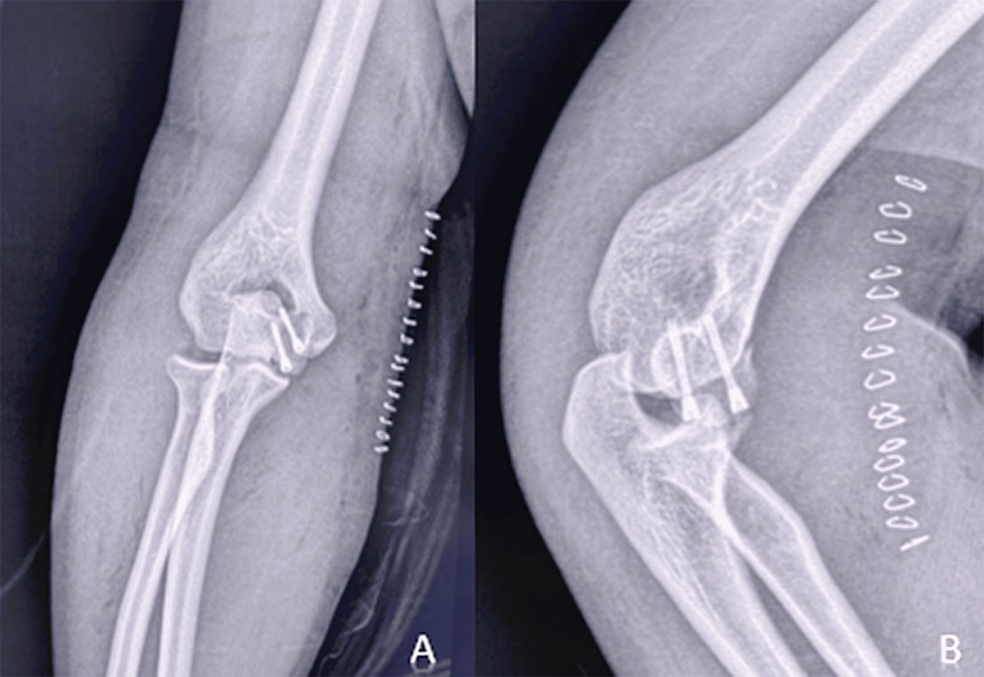
Postoperative frontal (A) and lateral (B) control X-rays showing the osteosynthesis in place

Immediately postoperatively, a standard radiograph of the elbow was performed (Figure 4), and passive and active range of motion exercises were initiated. The patient was discharged the following day with analgesics.

**Figure 5:**
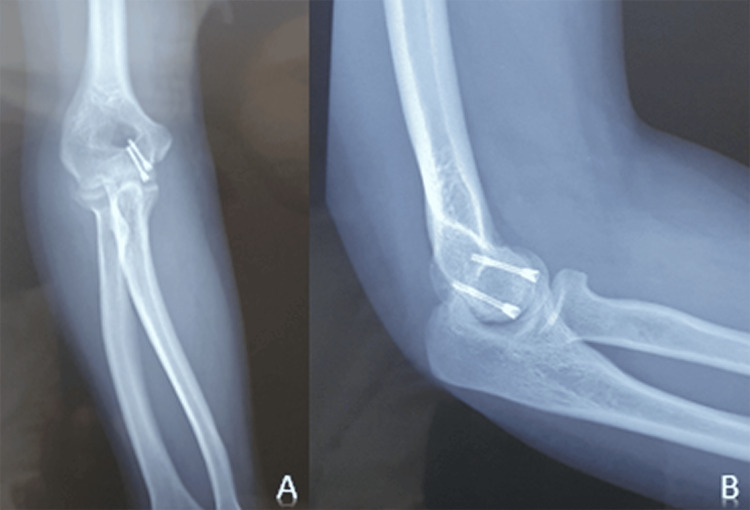
Follow-up standard radiograph in anteroposterior (A) and lateral (B) views showing fracture consolidation at two months

Currently, at two months postoperative, a standard X-ray shows bone consolidation (Figure 5), the patient reports no pain, and joint ranges of motion are as follows: pronation-supination is full, flexion is approximately 120°, and extension is limited to approximately 15°. The patient has resumed professional activities while continuing rehabilitation.

## Discussion

The trochlea is the articular surface of the distal humerus acting as a pulley on which the ulnohumeral joint moves [3].

An isolated trochlear fracture is rare [4]. What is even rarer is the partial character. Trochlear fractures are usually associated with fractures of the capitulum or medial condyle and/or elbow dislocation [5].

Our case is a partial internal fracture of the humeral trochlea associated with a small avulsion of the coronoid process without involvement of the capitulum or elbow dislocation or instability.

Guitton et al. reported that partial articular fractures of the distal humerus account for 3% to 4% of all fractures of the distal humerus. These partial articular fractures represent 15% of surgical indications, and 79% of these fractures involve both the capitulum and the trochlea [6].

Regarding the classification of capitellar fractures, type 1 (Hahn-Steinthal) involves the entire anterior capitulum, type 2 (Kocher-Lorenz) encompasses the entire anterior cartilaginous surface, and type 3 denotes a comminuted fracture. Additionally, type 4 indicates an extension into the lateral trochlea. In another classification, type 1 involves only the capitulum, type 2 includes both the capitulum and the lateral trochlea, and type 3 involves the entire distal articular surface with comminution. However, these two classifications do not include isolated anterior trochlear fractures, which should be considered type 4 due to their injury mechanisms, management principles, and different prognosis [7,8].

Among the various classifications of distal humeral articular fractures, type 3 of the Dubberley classification involves the trochlea, whereas those of the French Society of Orthopedic and Traumatological Surgery and Arbeitsgemeinschaft für Osteosynthesefragen/Orthopaedic Trauma Association do not define any type exclusively concerning the trochlea [8-10]. Our case is a fracture detaching approximately 50% of the medial border of the trochlea associated with a coronoid process avulsion type I according to the Regan and Morrey classification. Coronoid fractures of the distal humerus typically involve the capitulum and a variable portion of the trochlea [7]. Therefore, in the presence of any trochlear fracture, a thorough analysis of the capitulum is necessary.

Due to its deep location in the elbow, the trochlea is less exposed to direct trauma. Its injuries often occur through an indirect mechanism involving shearing forces. Trochlear fractures are rarely isolated and are usually associated with elbow dislocation, ligamentous injuries, capitellar fracture, radial head fracture, or olecranon fracture [1,8].

Functional complaints may be subtle, manifesting as a simple deficit in flexion or extension, either actively or passively, with preserved elbow contours [1]. Standard frontal radiographs may show irregularity of the articular surface or appear normal. On the lateral view, there may be a displaced semilunar appearance, directed upwards and forwards, which may erroneously suggest a capitellar fracture. Therefore, a CT scan is often requested [3]. Preoperative CT allows for a more detailed analysis of the fracture and detection of associated injuries [11]. CT scanning provides information on fragment size and displacement, aiding in therapeutic decision-making [5]. Three-dimensional reconstruction can be particularly useful, offering interobserver reproducibility [1,12,13]. As a result, the superposition of various fragments is no longer an obstacle, simplifying the choice of surgical approach [1].

Treatment is conservative for non-displaced fractures and is typically seen in elderly patients. There is no consensus on the duration of immobilization, but some suggest three weeks of posterior splinting [14-16]. In cases of displaced fractures, surgery is indicated. The medial or anteromedial approach is most commonly used in the literature. Several options are available for fixation, including Herbert screws, cancellous screws, or Kirschner wires. The choice depends primarily on the fragment size [5]. While wires stabilize the fragments, they do not offer as much compression as screws. The risk of osteonecrosis is low, even if the fragments have little or no muscular attachments [11,17]. The functional outcome of an isolated trochlear and/or capitellar fracture is better than that of a complex fracture, provided that the patient fully adheres to the rehabilitation protocol [8].

## Conclusions

Isolated partial fractures (without involvement of the capitulum) of the humeral trochlea are rare. In cases of displacement, we recommend open reduction, stable fixation, removal of small free arthrogenic fragments, and immediate postoperative active mobilization to avoid elbow stiffness. The functional prognosis is better compared to a fracture involving the humeral condyle.
